# Functional and structural alterations as diagnostic imaging markers for depression in *de novo* Parkinson’s disease

**DOI:** 10.3389/fnins.2023.1101623

**Published:** 2023-02-22

**Authors:** Hui Wang, Jianxia Xu, Miao Yu, Gaiyan Zhou, Jingru Ren, Yajie Wang, Huifen Zheng, Yu Sun, Jun Wu, Weiguo Liu

**Affiliations:** ^1^Department of Neurology, Lianyungang Hospital of Traditional Chinese Medicine, Lianyungang Affiliated Hospital of Nanjing University of Chinese Medicine, Lianyungang, China; ^2^Department of Neurology, Dushu Lake Hospital Affiliated to Soochow University, Suzhou, China; ^3^Department of Neurology, The Affiliated Brain Hospital of Nanjing Medical University, Nanjing, China; ^4^Department of Neurology, Geriatric Hospital of Nanjing Medical University, Nanjing, China; ^5^International Laboratory of Children Medical Imaging Research, School of Biological Science and Medical Engineering, Southeast University, Nanjing, China; ^6^Department of Clinical Laboratory, The Affiliated Brain Hospital of Nanjing Medical University, Nanjing, China

**Keywords:** Parkinson’s disease, depression, resting state functional magnetic resonance imaging, structural magnetic resonance imaging, differential diagnosis

## Abstract

**Background:**

Depression in Parkinson’s disease (PD) is identified and diagnosed with behavioral observations and neuropsychological measurements. Due to the large overlaps of depression and PD symptoms in clinical manifestations, it is challenging for neurologists to distinguish and diagnose depression in PD (DPD) in the early clinical stage of PD. The advancement in magnetic resonance imaging (MRI) technology provides potential clinical utility in the diagnosis of DPD. This study aimed to explore the alterations of functional and structural MRI in DPD to produce neuroimaging markers in discriminating DPD from non-depressed PD (NDPD) and healthy controls (HC).

**Methods:**

We recruited 20 DPD, 37 NDPD, and 41 HC matched in age, gender, and education years. The patients’ diagnosis with PD was *de novo*. The differences in regional homogeneity (ReHo), voxel-wise degree centrality (DC), cortical thickness, cortical gray matter (GM) volumes, and subcortical GM volumes among these groups were detected, and the relationship between altered indicators and depression was analyzed. Moreover, the receiver operating characteristic (ROC) analysis was performed to assess the diagnostic efficacy of altered indicators for DPD.

**Results:**

Compared to NDPD and HC, DPD showed significantly increased ReHo in left dorsolateral superior frontal gyrus (DSFG) and DC in left inferior temporal gyrus (ITG), and decreased GM volumes in left temporal lobe and right Amygdala. Among these altered indicators, ReHo value in left DSFG and DC values in left ITG and left DSFG were significantly correlated with the severity of depression in PD patients. Comparing DPD and NDPD, the ROC analysis revealed a better area under the curve value for the combination of ReHo value in left DSFG and DC value in left ITG, followed by each independent indicator. However, the difference is not statistically significant.

**Conclusion:**

This study demonstrates that both functional and structural impairments are present in DPD. Among them, ReHo value of left DSFG and DC value of left ITG are equally well suited for the diagnosis and differential diagnosis of DPD, with a combination of them being slightly preferable. The multimodal MRI technique represents a promising approach for the classification of subjects with PD.

## 1. Introduction

Parkinson’s disease (PD) is one of the most prevalent neurodegenerative diseases, displaying motor and non-motor symptoms (Poewe et al., 2017). Among non-motor symptoms of PD, depression is one of the most common and debilitating, aggravating patient disability, reducing the quality of life, and increasing the burden on caregivers (Schapira et al., 2017). Depression can occur throughout the PD course, especially in the early stages, and even precede motor symptoms in the prodromal phase (Schrag et al., 2015). The etiology and pathogenesis of depression in PD (DPD), according to the neurotransmitter theory, involves multiple neurotransmitter dysfunction, including dopamine, serotonin, and norepinephrine. The involvement of raphe nuclei (serotonin) and locus coeruleus (norepinephrine) at Braak stage 2, and midbrain (dopamine) at Braak stage 3 may indicate the high incidence and the specific pathological mechanism of depression in the early stage of Parkinson’s disease (Braak et al., 2003, 2004; Schapira et al., 2017). Therefore, a better understanding of the specific pathological damages of DPD at an early stage is crucial for early diagnosis and treatment.

Currently, DPD is identified and diagnosed by behavioral observations and neuropsychological measurements. Due to the large overlap of depression and PD symptoms in clinical manifestations, it is challenging for neurologists to distinguish and diagnose DPD in the early clinical stage of PD (Assogna et al., 2020). Early and accurate diagnosis is the primary prerequisite for timely and effective interventions. Central to this problem is the search for more precise and measurable diagnostic tools that can distinguish DPD from early-stage PD.

The advancement in magnetic resonance imaging (MRI) technology provides insights into abnormal brain function and structure, and potential clinical utility in DPD, from diagnosis to therapeutic interventions. Studies based on the functional metrics, the amplitude of low-frequency fluctuation (ALFF), and regional homogeneity (ReHo), demonstrated that the regional brain dysfunction in the prefrontal-limbic system, basal ganglia, and default network contributes to DPD (Wen et al., 2013; Sheng et al., 2014; Hu et al., 2015a; Huang et al., 2015). Considering functional integration, studies using seed-based functional connectivity analysis illustrated that the neural mechanism of DPD involves the reduced connectivity of the limbic-cortical circuit and the increased connectivity between specific limbic areas and temporal cortex (Luo et al., 2014; Sheng et al., 2014; Hu et al., 2015a,b). Liao et al. (2021) performed the data-driven method termed degree centrality (DC) and revealed that the prefrontal lobe, limbic system, and basal ganglia are key nodes changed in DPD. About structural MRI (sMRI) studies, converging pieces of evidence had shown significant gray matter (GM) volume/cortical thickness abnormalities in DPD, including frontal, temporal, precuneus, cingulate gyrus, as well as hippocampal, para-hippocampal cortices, and amygdala (van Mierlo et al., 2015; Luo et al., 2016; Chagas et al., 2017; Hanganu et al., 2017; Goto et al., 2018). Overall, the above neuroimaging findings allow us to propose that the occurrence and development of DPD are due to alterations in the structure and function of specific brain regions, which may be used as markers of the ongoing neurodegenerative process to facilitate the early diagnosis of this disease.

Exploring these multimodal MRI features contributes to understanding the neurophysiological mechanisms of DPD and aids in the differential diagnosis. However, the previous results indicated no uniform prediction model for the diagnosis, but a wide range of structural and functional brain alterations associated with DPD (Valli et al., 2019). In addition, previous studies were limited to the abnormalities of specific brain regions based on different hypotheses, under different MRI imaging modes, so the comparability of the results is poor, the exploration of the whole brain structure and function is not comprehensive, and even the results are affected by selection bias. Moreover, rare studies have excluded medications as a potential confounding factor. Studies reported the effects of medication therapy on brain structure and function (Jung et al., 2014; Yan et al., 2019). Even if some studies recruited patients who stopped taking drugs for a period before enrollment (Dan et al., 2017), the altered brain patterns might be associated with the chronic effect of medications rather than with the influence of neurological impairment. To overcome these limitations, we recruited *de novo* unmedicated PD patients and applied a multivariate approach to the analysis of data-driven multimodal MRI metrics to explore the potential value of the structural and functional features in differentiating DPD from non-depressed PD (NDPD) and healthy controls (HC).

We hypothesized that (1) early, untreated DPD patients had alterations in multimodal MRI indicators, (2) the abnormal indicators would be related to the clinical depression score, and (3) the combination of the altered indicators might constitute a potential neuroimaging marker that could be applied to distinguish *de novo* DPD from NDPD or HC with high accuracy.

## 2. Materials and methods

### 2.1. Subjects

The study was approved by the Medical Research Ethics Committee of Nanjing Brain Hospital. Written informed consent according to the “Declaration of Helsinki” principles was obtained from all study participants. *De novo* untreated PD patients were recruited from the Movement Disorder Clinic of the Affiliated Brain Hospital of Nanjing Medical University. The diagnosis was made by an experienced neurologist according to the United Kingdom Parkinson’s Disease Society Brain Bank diagnostic criteria (Hughes et al., 1992). Depression was diagnosed by a senior psychiatrist according to the Diagnostic and Statistical Manual of Mental Disorders, Fifth Edition. Meanwhile, a 17-item Hamilton Depression Rating Scale (HAMD-17) score higher than 14 was required. Diagnoses of PD and depression were completed on the same day during the study recruitment. Patients were excluded if they had: (1) anti-Parkinsonism or antidepressants medications, or other psychiatric therapy before enrollment; (2) a history of cerebrovascular disorders, head injury, neurological surgery, or other neurologic diseases; (3) a history of any psychiatric diseases; (4) Dementia, the score of Mini-Mental State Examination (MMSE), weighted by Chinese education, < 17 for illiterate subjects, < 20 for grade-school literate, and < 24 for junior high school and higher education literate (Li et al., 2016); (5) history of severe chronic diseases; (6) Contraindications for MRI scanning or MRI showing cerebral infarction, obvious brain atrophy or other intracranial lesions. (7) Incomplete clinical information. All patients were followed for at least one year after clinical evaluation and MRI scans to confirm the diagnosis according to the evolution of the disease and response to dopaminergic therapy. Healthy controls (HC), matched with the PD in age, gender, and years of education, were recruited from the health examination center of the Affiliated Brain Hospital of Nanjing Medical University. According to the above criteria, this study included 21 DPD, 39 NDPD, and 43 HC. However, one DPD, two NDPD, and two HC subjects were excluded due to excessive head motion (see below). This study eventually included 20 DPD, 37 NDPD, and 41 HC, who participated in the subsequent statistical analysis.

### 2.2. Measurement of clinical characteristics

Demographic and clinical details were obtained during the baseline visits. Motor severity and disease severity were evaluated by the motor section of the Unified Parkinson’s Disease Rating Scale and the Hoehn and Yahr staging scale. The severity of depression was quantified using HAMD-17, while cognitive impairment evaluation was assessed based on MMSE. All assessments and fMRI scans were performed on the same day during the study recruitment.

### 2.3. Acquisition of MRI data

MRI was performed on the same 3T MRI scanner (Siemens, Verio, Germany). All participants were instructed to lay supine, remain as still as possible, close their eyes, and remain awake without thinking anything during the scan. Foam pads and a standard birdcage head coil were used to minimize head movement. Axial anatomical images were acquired using a T1 fluid-attenuated inversion recovery sequence for image registration and functional localization with the following parameters: repetition time = 2,530 ms; echo time = 3.34 ms; flip angle = 7 degrees; matrix = 256 × 192; field of view = 256 × 256 mm; slice thickness/gap = 1.33/0.5 mm; bandwidth = 180 HZ/PX; 128 slices covered the whole brain. Functional images were subsequently collected in the same slice orientation with a gradient-recalled echo-planar imaging pulse sequence, which included 240 volumes. The parameters were: repetition time = 2,000 ms; echo time = 30 ms; flip angle = 90 degrees; matrix = 64 × 64; field of view = 220 × 220 mm; thickness/gap = 3.5/0.6 mm; bandwidth = 2,232 HZ/PX.

### 2.4. Preprocessing of resting-state fMRI

All resting-state fMRI (rs-fMRI) data were preprocessed with the Data Processing and Analysis toolbox for (Resting-State) Brain Imaging (DPABI Version 4.3^1^) (Yan et al., 2016), based on the MATLAB 2013b platform.^2^

First, the first ten volumes of each rest functional section were excluded for signal equilibrium and to allow participants adaptation to the scanning environment. Subsequently, the remaining images were corrected for slice timing using the middle slice as a reference and then realigned to remove head motion. Second, the structural images were coregistered to the mean functional images after realignment. The transformed structural images were then segmented into gray matter, white matter, and CSF. Based on the segmented images, the Diffeomorphic Anatomical Registration Through Exponentiated Lie Algebra (DARTEL) algorithm (Ashburner and Friston, 2009) was used to compute transformations from individual native space to the standard Montreal Neurological Institute (MNI) space. Then, several nuisance covariates, including the Friston-24 head motion parameters (Friston et al., 1996), linear and quadratic trends, and CSF and white matter signals, were regressed to minimize the motion artifact and improve the signal-noise ratio of functional volumes. The resulting functional images were then normalized into the MNI space using the normalization parameters estimated in DARTEL and resampled to 3 × 3 × 3 mm^3^ voxel size, and temporal band-pass filtered (0.01–0.08 Hz). To minimize the potential effects of head motion, we excluded participants with a mean framewise displacement (FD) > 0.5 mm or head motions exceeding 3.0 mm translation or 3.0° rotation (Navalpotro-Gomez et al., 2020). Notably, the GM volume map of each subject was also extracted from DARTEL for use as a covariate in the voxel-based analyses of the fMRI data.

### 2.5. FMRI indicators measurement

FMRI indicators were calculated using DPABI Version 4.3. Kendall’s coefficient of concordance was calculated at each voxel to establish similarities between the time series of each specific voxel and its 26 adjacent voxels. Normalization was performed by dividing Kendall’s coefficient of concordance among each voxel by the average Kendall’s coefficient of concordance of the whole brain to reduce the influence of individual variations.

As a voxel-wise measurement for the whole-brain functional connectivity, DC could reflect the global functional connections of brain hubs (Zuo et al., 2012). For each voxel, the time course was extracted and correlated with every other voxel in the brain. A whole-brain connectivity matrix for each subject was constructed and binarized with the correlation threshold being set at *r* > 0.25 before counting the number of connections to generate voxel-wise DC to eliminate possible spurious correlations arising from noises (Buckner et al., 2009; Wang et al., 2020). The matrix was transformed into a *z*-score matrix using Fisher’s r-to-z transformation to improve the normality.

Finally, the calibrated ReHo maps and the resulting DC maps were spatially smoothed using a Gaussian kernel with a full-width half maximum (FWHM) of 6 mm (Li et al., 2017; Wang et al., 2020).

### 2.6. Preprocessing of sMRI

Structural T1 images were analyzed using FreeSurfer (version 6.0.0^3^) to perform cortical modeling and volumetric segmentation. The standard processing procedures were performed separately on each cerebral hemisphere and included (1) motion correction and conform; (2) correction of signal strength non-uniformities caused by magnetic field inhomogeneities; (3) removal of no-brain issue (skull stripping); (4) affine registration to the Talairach atlas and segmentation of the subcortical white matter and deep gray matter structures. (5) tessellation of the gray-to-white and gray-to-cerebrospinal fluid surface boundaries; (6) automatic correction of topology defects; (7) surface deformation for optional placement of the gray-to-white and gray-to-CSF boundaries, two researchers blinded to the participants performed the initial visual inspection of the segmentation and minor manual corrections to the segmentation as needed; (8) smoothing with a 10 mm FWHM Gaussian smoothing kernel; (9) surface inflation and registration to a spherical atlas for intersubject matching of cortical folding patterns; (10) cortical parcellations were based on the PALS-B12 atlas (Van Essen, 2005). For each subject, per hemisphere, FreeSurfer parcellated five cortical regions (frontal, parietal, limbic, temporal, and occipital lobe) based on the PALS-B12 atlas and seven subcortical regions (nucleus accumbens, amygdala, caudate, hippocampus, pallidum, putamen, and thalamus) (Fischl et al., 2002). The regional cortical thicknesses and volumes, subcortical GM volumes, and total intracranial volume (TIV) were extracted from the reconstructed brain images in the standard brain space.

### 2.7. Statistical analysis

Statistical analyses of the demographic, clinical variables, mean FD, and the volume of interest (VOI) based sMRI data were performed using the Statistical Product and Service Solutions version 19.0. After checking for normal distribution and homogeneity of variance within the data, a one-way analysis of variance or Kruskal-Wallis test was performed on the continuous data with three levels of groups. The GLM was conducted to compare the differences of the sMRI data across the three groups, with age, years of education, MMSE, and TIV as covariates, Bonferroni corrected. A *post-hoc* test, corrected by a Bonferroni test, was then conducted for pairwise comparison in each analysis. Differences between the two group levels were calculated by a two-sample *t*-test or a Mann-Whitney *U*-test. At the same time, the chi-square test was used for the analysis of categorical variables. A *p*-value < 0.05 was considered statistically significant.

Differences between groups in rs-fMRI indicators and the GM volume maps extracted from DARTEL were analyzed in the DPABI statistical analysis module. A voxel-based one-way analysis of covariance (ANCOVA) within GM mask was performed to compare the differences among three groups, followed by two-sample *t*-tests within the result masks from ANCOVA. All statistical analyses were corrected according to the Gaussian random field (GRF) theory (voxel *p*-value < 0.001, cluster *p*-value < 0.05) using MMSE, years of education, mean FD, and GM volume as covariates. All significant results were reported in the DPABI viewer module, including the peak MNI coordinates, the peak MNI coordinate regions (according to the Anatomical Automatic Labeling atlas), and the size of each cluster.

To investigate the relationship between neuroimaging abnormalities and the severity of depression, the significantly altered MRI indicators were extracted for a partial correlation analysis with HAMD-17 in PD patients, using MMSE scores as covariates, Bonferroni corrected.

ROC curves were analyzed based on the significantly altered MRI indicators between DPD and NDPD, DPD and HC, to assess their value in the diagnosis and differentiation of DPD. First, each significantly altered neuroimaging indicator between DPD and NDPD, DPD and HC were used as independent variables to establish a binary logistic regression model, respectively. The stepwise backward selection was used to identify indicators that were independent predictors of DPD. The exclusion significance level was set to 0.1. At the same time, we obtained the prediction probability for the combination of the above-selected indicators. Then, we computed the areas under a ROC curve (AUC) of each predictor which survived the logistic regression analysis and the AUC of the combined factor. The 95% confidence intervals were calculated using the pROC software package with bootstrapping (*n* = 10,000 iterations). We calculated the highest Youden index and evaluated the sensitivity and specificity of each and the combined factor. The diagnostic performance of the predictors and the combined factor was compared using the De Long test in Medcalc software.^4^

## 3. Results

### 3.1. Demographic and clinical characteristics

The statistical analysis included data from 98 subjects: 20 DPD, 37 NDPD, and 41 HC. There were no significant differences between the three groups in terms of age, gender, and years of education. No significant differences in disease duration, UPDRS-III, and Hoehn and Yahr stage were found between the two PD groups. However, DPD had significantly lower MMSE scores than HC, but no difference was found between DPD and NDPD, NDPD, and HC. Significant differences existed among the HAMD-17 scores of the three groups (Table 1).

**TABLE 1:** Demographic and clinical characteristics of three groups, including DPD, NDPD, and HC.

Groups	DPD (*n* = 20)	NDPD (*n* = 37)	HC (*n* = 41)	*P*-value	*Post hoc P*-value
Age, year	59.2 ± 6.3	58.7 ± 9.3	60.1 ± 6.2	0.721*	
Gender, male/female	8/12	20/17	21/20	0.586 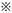	
Education, year	9.0 ± 3.3	9.3 ± 3.5	10.9 ± 3.5	0.053*	
PD duration time, year	2.0 (1.0,3.0)	1.0 (1.0,2.5)	–	0.360◆	
UPDRS-III	28.7 ± 8.9	24.9 ± 8.8	–	0.132♢	
H&Y,1.0/1.5/2.0/2.5/3.0	4/7/5/3/1	10/13/12/2/0	–	0.488^△^	
MMSE	27.00 (25.25, 28.75)	29.00 (27.00, 29.00)	29.00 (28.00, 30.00)	**0.009**◆	DPD < NDPD: *P* = 0.171 DPD < HC: *P* = **0.007** NDPD < HC: *P* = 0.539
HAMD-17	15.50 (14.00, 19.25)	3.00 (2.00, 5.00)	0.00 (0.00, 3.00)	**0.000**◆	DPD > NDPD: *P* = **0.000** DPD > HC: *P* = **0.000** NDPD > HC: *P* = **0.000**

Data are presented as mean ± standard deviation, median (lower quartile, upper quartile) for continuous variables or frequencies for categorical ones. * *P*-values were obtained using one-way ANOVA tests; 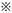 *P*-values were obtained using chi-square tests; ◆*P*-values were obtained using Mann-Whitney-*U* tests or Kruskal-Wallis H tests; ♢*P*-values were obtained by two-sample *T*-test; ^△^*P*-values were obtained by Fisher’s Exact Test. *P*-values < 0.05 was considered statistically significant. Bold indicates statistically significant difference. UPDRS-III, the Unified Parkinson’s Disease Rating Scale (motor part III); H&Y, Hoehn and Yahr staging; MMSE, Mini-Mental State Exam; HAMD-17, the 17-item Hamilton Depression Rating Scale.

### 3.2. Univariate analysis of voxel-based indicators

There was no significant difference in mean FD (Kruskal-Wallis *H* tests *H* = 3.721, *p*-value = 0.156) and the voxel-based GM volume maps of the three groups, indicating that the head motion and cortical atrophy did not affect the fMRI findings.

**FIGURE 1:**
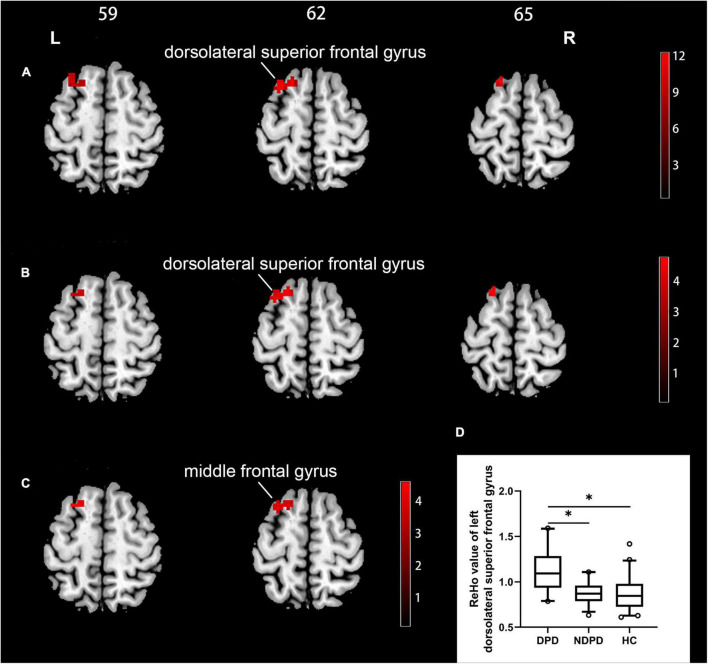
Brain regions exhibiting significant differences in ReHo. **(A)** Group comparisons of the ReHo among DPD, NDPD, and HC groups. The color bar represents the *F* value of ANCOVA. **(B)** *Post hoc* analysis of DPD vs. NDPD. The color bar indicates the *T*-value from the two-sample *t*-test within the ANCOVA result masks. **(C)** *Post hoc* analysis of DPD vs. HC. The color bar indicates the *T*-value from the two-sample *t*-test within the ANCOVA result masks. **(D)** Box-and-whiskers plot for the ReHo value of the left dorsolateral superior frontal gyrus in DPD, NDPD, and HC groups. The boxes display the median and the 25th and 75th quartiles, whiskers showing the 5th and the 95th percentile. The outlier is shown as a data point outside of the box. *Post hoc P-value < 0.05, Bonferroni corrected. L, left; R, right.

A significant group difference among the three groups in ReHo was detected by ANCOVA analysis in the left dorsolateral superior frontal gyrus (DSFG)/the left middle frontal gyrus. Compared to NDPD, DPD showed a significant increase of ReHo in left DSFG. Compared to HC, DPD showed a significant increase of ReHo in the left middle frontal gyrus. There is no significant difference between NDPD and HC (Table 2 and Figure 1).

**TABLE 2:** The differences of ReHo among three groups.

Cluster location	BA	Clusters size (ml)	Coordinates MNI			*F*/*T* value
			***X***	***Y***	***Z***	
***ANCOVA***						
Left superior frontal gyrus, dorsolateral/left middle frontal gyrus	8	0.999	−18	21	63	12.298
***Post-hoc tests: DPD* > *NDPD***						
Left superior frontal gyrus, dorsolateral	8	0.756	−18	21	63	4.745
***Post-hoc tests: DPD* > *HC***						
Left middle frontal gyrus	8	0.594	−24	18	60	4.588

BA, Brodmann area; MNI, Montreal Neurological Institute.

**FIGURE 2:**
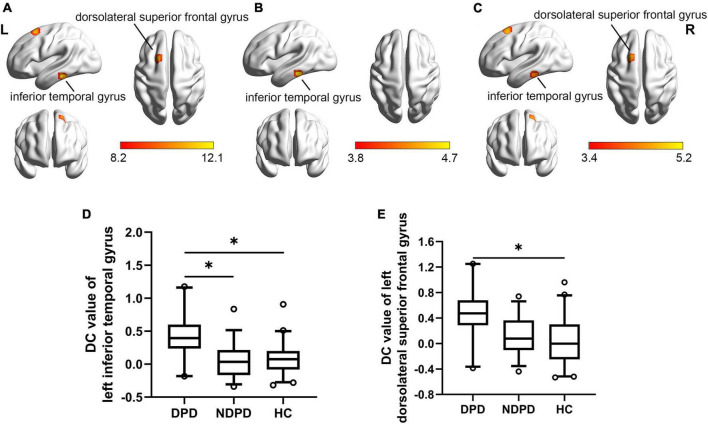
Brain regions exhibiting significant differences in DC. **(A)** Group comparisons of the DC among DPD, NDPD, and HC groups. The color bar represents the *F* value of ANCOVA. **(B)** *Post hoc* analysis of DPD vs. NDPD. The color bar indicates the *T*-value from the two-sample *t*-test within the ANCOVA result masks. **(C)** *Post hoc* analysis of DPD vs. HC. The color bar indicates the *T*-value from the two-sample *t*-test within the ANCOVA result masks. **(D,E)** Box-and-whiskers plots for the DC value of left inferior temporal gyrus and left dorsolateral superior frontal gyrus in DPD, NDPD, and HC groups. The boxes display the median and the 25th and 75th quartiles, whiskers showing the 5th and the 95th percentile. The outlier is shown as a data point outside of the box. *Post hoc P-value < 0.05, Bonferroni corrected. L, left; R, right.

The DC of the left inferior temporal gyrus (ITG) and left DSFG displayed significant differences among the three groups. Compared to NDPD, DPD exhibited a significant DC increase in left ITG. Meanwhile, the DC of the left ITG and left DSFG dramatically increased in DPD compared to HC. There is no significant difference between NDPD and HC (Table 3 and Figure 2).

**TABLE 3:** The differences of DC among three groups.

Cluster location	BA	Clusters size (ml)	Coordinates MNI			*F*/*T* value
			***X***	***Y***	***Z***	
***ANCOVA***						
Left inferior temporal gyrus	20	0.513	−63	−33	−21	12.068
Left superior frontal gyrus, dorsolateral	8	0.729	−21	15	60	10.852
***Post-hoc tests: DPD* > *NDPD***						
Left inferior temporal gyrus	20	0.513	−63	−33	−21	4.645
***Post-hoc tests: DPD* > *HC***						
Left inferior temporal gyrus	20	0.459	−69	−36	−21	5.178
Left superior frontal gyrus, dorsolateral	8	0.567	−21	15	57	4.664

BA, Brodmann area; MNI, Montreal Neurological Institute.

### 3.3. Univariate analysis of VOI-based indicators

First, there was no significant difference among the three groups in TIV extracted from the VOI segmentation, indicating that TIV had little effect on the statistical results of structural data (Table 4).

**TABLE 4:** The differences of cortical GM volumes of PALS_B12 lobes among three groups.

Regions	DPD (*n* = 20) (cm^3^)	NDPD (*n* = 37) (cm^3^)	HC (*n* = 41) (cm^3^)	*F*	*P*-value	*Post hoc P*-value
Left frontal	69.6 ± 8.1	70.7 ± 8.5	70.1 ± 6.5	0.632	0.534	
Left parietal	40.3 ± 5.3	40.2 ± 5.6	40.6 ± 4.6	0.573	0.566	
Left limbic	13.1 ± 1.3	13.4 ± 1.3	13.2 ± 1.7	0.993	0.374	
Left temporal	51.93 ± 3.5	54.7 ± 5.7	54.2 ± 5.7	5.827	**0.004**	**DPD** < **NDPD: *P* = 0.006** **DPD** < **HC: *P* = 0.009** NDPD > HC: *P* = 1.000
Left occipital	30.8 ± 3.4	31.6 ± 3.5	32.4 ± 3.9	6.952	**0.002**	DPD < NDPD: *P* = 0.201 **DPD** < **HC: *P* = 0.001** NDPD < HC: *P* = 0.069
Right frontal	71.8 ± 8.0	73.0 ± 9.0	71.6 ± 6.8	0.724	0.488	
Right parietal	35.8 ± 5.4	35.9 ± 5.0	36.5 ± 3.5	0.754	0.473	
Right limbic	13.1 ± 1.4	13.1 ± 1.2	13.1 ± 1.6	0.506	0.605	
Right temporal	54.9 ± 3.7	57.1 ± 5.2	56.7 ± 5.6	4.505	0.014	
Right occipital	32.4 ± 3.2	33.4 ± 4.3	33.5 ± 3.8	3.274	0.042	
TIV	1588.3 ± 163.0	1568.4 ± 144.7	1552.4 ± 181.4	0.328	0.721	

All results of generalized linear models were corrected by Bonferroni for multiple-comparison (*P*-value < 0.05/10). All *post hoc* analysis were corrected by Bonferroni test. Bold indicates statistically significant difference. TIV, total intracranial volume.

**FIGURE 3:**
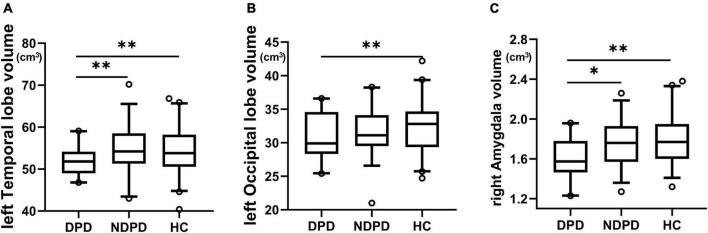
Box-and-whiskers plots for brain regions exhibiting significant differences in GM volumes. **(A)** The left temporal lobe, **(B)** the left occipital lobe, **(C)** the right amygdala. The boxes display the median and the 25th and 75th quartiles, whiskers showing the 5th and the 95th percentile. The outlier is shown as a data point outside of the box. *Post hoc P-value < 0.05, **Post hoc P-value < 0.01, Bonferroni corrected.

The ANOVA analysis showed significantly changed cortical and subcortical GM volumes across three groups, including the left temporal lobe, the left occipital lobe (Bonferroni corrected, *p*-value < 0.05/10), and the right Amygdala (Bonferroni corrected, *p*-value < 0.05/14). Compared to NDPD, DPD showed significantly decreased GM volumes in the left temporal lobe and right Amygdala. Compared to HC, DPD showed significantly decreased GM volumes in the left temporal lobe, left occipital lobe, and right Amygdala (Tables 4, 5, and Figure 3). There was no significant difference in cortical thickness among the three groups (Supplementary Table 1).

**TABLE 5:** The differences of subcortical GM volumes among three groups.

Regions	DPD (*n* = 20) (cm^3^)	NDPD (*n* = 37) (cm^3^)	HC (*n* = 41) (cm^3^)	*F*	*P*-value	*Post hoc P*-value
Left thalamus	6.59 ± 0.76	6.63 ± 0.76	6.54 ± 0.71	0.093	0.912	
Left caudate	3.14 ± 0.40	3.27 ± 0.41	3.17 ± 0.47	0.916	0.404	
Left putamen	4.66 ± 0.58	4.90 ± 0.63	4.70 ± 0.67	2.022	0.138	
Left pallidum	1.99 ± 0.21	2.11 ± 0.25	1.99 ± 0.25	3.670	0.029	
Left hippocampus	3.80 ± 0.27	3.88 ± 0.46	3.93 ± 0.34	2.768	0.068	
Left amygdala	1.46 ± 0.17	1.59 ± 0.25	1.56 ± 0.29	2.305	0.106	
Left accumbens	0.48 ± 0.10	0.48 ± 0.09	0.48 ± 0.09	0.014	0.986	
Right thalamus	6.56 ± 0.77	6.63 ± 0.74	6.48 ± 0.76	0.320	0.727	
Right caudate	3.22 ± 0.45	3.33 ± 0.49	3.19 ± 0.47	0.928	0.399	
Right putamen	4.73 ± 0.81	4.91 ± 0.73	4.79 ± 0.70	0.751	0.475	
Right pallidum	1.88 ± 0.26	2.0 ± 0.28	1.89 ± 0.28	2.446	0.092	
Right hippocampus	4.08 ± 0.29	4.13 ± 0.44	4.18 ± 0.38	1.740	0.181	
Right amygdala	1.61 ± 0.20	1.75 ± 0.22	1.78 ± 0.25	6.854	**0.002**	**DPD** < **NDPD: *P* = 0.010** **DPD** < **HC: *P* = 0.001** NDPD < HC: *P* = 1.000
Right accumbens	0.50 ± 0.10	0.52 ± 0.10	0.51 ± 0.08	0.125	0.882	

All results of generalized linear models were corrected by Bonferroni for multiple-comparison (*P*-value < 0.05/14). All *post hoc* analysis were corrected by Bonferroni test. Bold indicates statistically significant difference.

### 3.4. Correlation analysis

**FIGURE 4:**
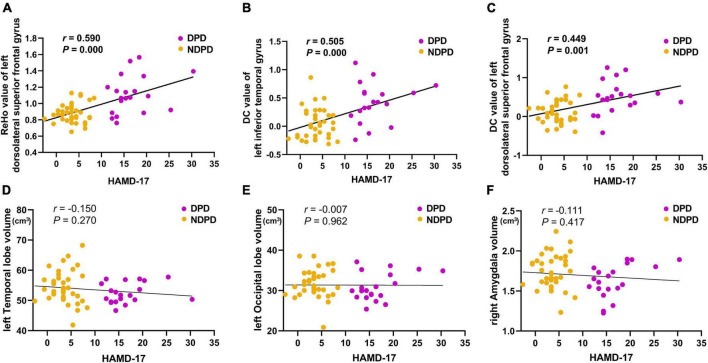
The correlations of **(A)** the ReHo value of left dorsolateral superior frontal gyrus, **(B)** the DC value of left inferior temporal gyrus, **(C)** the DC value of left dorsolateral superior frontal gyrus, **(D)** the left temporal lobe volume, **(E)** the left occipital volume, and **(F)** the right amygdala volume with the HAMD-17 scores. MMSE was used as the covariate of results (Bonferroni corrected, *p* < 0.05/6).

The ReHo values of left DSFG in patient groups showed a positive correlation with HAMD-17 scores (*r* = 0.590, *p*-value = 0.000). In addition, the DC values of left ITG (*r* = 0.505, *p*-value = 0.000) and left DSFG (*r* = 0.449, *p*-value = 0.001) were also significantly positively associated with HAMD-17 scores in patient groups (Bonferroni corrected, *p* < 0.05/6). However, no significant correlation was recorded between the other altered indicators and the HAMD-17 score (Figure 4).

### 3.5. Diagnosis of DPD using the ROC analysis

**FIGURE 5:**
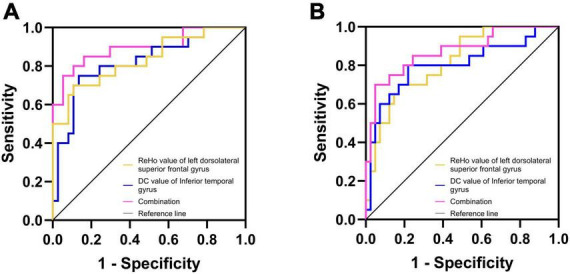
Diagnosis and differentiation of DPD based on ROC analysis. **(A)** ROC curve showing the classification of DPD and NDPD; **(B)** ROC curve showing the classification of DPD and HC.

In DPD and NDPD groups, the binary logistic regression models with DPD as the dependent variables and each significantly altered MRI indicator as the independent variable showed that the ReHo value of left DSFG (*p*-value = 0.003) and the DC value of left ITG (*p*-value = 0.006) were independent predictors of DPD. Further ROC analysis was carried out to evaluate the diagnostic power of the above independent predictors and their combination. The AUC value of the ReHo value of left DSFG was 0.834 (95% CI 0.710 ∼ 0.938), *p*-value < 0.001 with sensitivity = 70.0%, specificity = 89.2%. The AUC value of the DC value of left ITG was 0.820 (95% CI 0.688 ∼ 0.928), *p*-value < 0.001 with sensitivity = 75.0%, specificity = 86.5%. A more optimized classification model was based on a combination of the above independent predictors. The AUC value of combined factor was 0.896 (95% CI 0.791 ∼ 0.978), *p*-value < 0.001 with the sensitivity = 75.0%, specificity = 94.6% (Figure 5A). The De Long test showed no differences in the diagnostic performance between the ReHo value of left DSFG and the DC value of left ITG (*D* = 0.014, 95% CI −0.141∼0.168, *p*-value = 0.864), the ReHo value of left DSFG and the combined factor (*D* = 0.062, 95% CI −0.017∼0.142, *p*-value = 0.126), and the DC value of left ITG and the combined factor (*D* = 0.076, 95% CI −0.015∼0.167, *p*-value = 0.103).

The same analysis was performed to verify the possible diagnostic and differential diagnostic values of the significantly altered indicators between the DPD and HC groups. Results showed the ReHo value of left DSFG (*p*-value = 0.004) and the DC value of left ITG (*p*-value = 0.004) were independent predictors of DPD. A ROC analysis was performed. The AUC value for the ReHo values of left DSFG was 0.817 (95% CI 0.699 ∼ 0.915), *p*-value < 0.001 with sensitivity = 70.0%, specificity = 82.9%. The AUC value of the DC value of left ITG was 0.800 (95% CI 0.659 ∼ 0.918), *p*-value < 0.001 with sensitivity = 80.0%, specificity = 78.0%. The AUC value of the combined factor was 0.873 (95% CI 0.766 ∼ 0.960), *p*-value < 0.001 with sensitivity = 70.0%, specificity = 95.1% (Figure 5B). The De Long test showed no differences in the diagnostic performance between the ReHo value of left DSFG and the DC value of left ITG (*D* = 0.017, 95% CI −0.143∼0.178, *p*-value = 0.835), the ReHo value of left DSFG and the combined factor (*D* = 0.056, 95% CI −0.033∼0.145, *p*-value = 0.215), and the DC value of left ITG and the combined factor (*D* = 0.073, 95% CI −0.012∼0.158, *p*-value = 0.092).

## 4. Discussion

The current study aimed to systematically investigate the brain functional and structural MRI changes related to DPD within the whole-brain range and to explore the auxiliary value of the altered MRI indicators in the clinical diagnosis of DPD. First, we confirmed the brain functional and structural impairments regarding ReHo, DC, and GM volumes in DPD. Second, the ReHo value of left DSFG and the DC values of left ITG and left DSFG are significantly positively associated with the depression score. Third, the ReHo value of left DSFG, the DC value of left ITG, and the combined two indicators were identified as potential markers in DPD for their high sensitivity and specificity in distinguishing DPD from NDPD and HC. Moreover, the combined factor displayed a slightly stronger trend rather than significant efficacy in the differential diagnosis of DPD. However, the specificity of the combination was higher than each indicator.

### 4.1. Functional alterations in DPD

A growing body of fMRI research has investigated the dysfunction of ITG in patients with depression. Analyses of network homogeneity, ReHo, and ALFF found that depressed patients have decreased network homogeneity in the left ITG (Yan et al., 2021), increased ReHo (Li et al., 2018), and lower ALFF (Guo et al., 2013) in the ITG. Overall, it is reasonable to speculate that abnormal function in the ITG may be universal and distinct in depression and that this region may play a crucial role in the pathophysiology of depression. However, studies on DPD seem to report few findings involving ITG. Recent work on DPD constructed a functional brain network by parcellating the whole brain into 90 regions using the atlas of automated anatomical labels. The results showed that the right ITG was the hub region in the HC and NDPD but not in the DPD, and the DPD group had weaker connections between the temporal-occipital visual cortex and the prefrontal-limbic network (Qiu et al., 2021). Another study based on DC reported an increased DC value of the left superior temporal gyrus in DPD, compared to NDPD patients (Liao et al., 2021). Therefore, it can be speculated that the function of the temporal lobe altered in DPD compared to NDPD and HC, which was also confirmed by our study. However, inconsistent with the above studies, we observed a significant increase in the DC value of the left ITG in DPD patients compared to the other two groups, which was significantly positively correlated with the depression scores. We could not compare them directly, not only because of the different methods of brain network construction and diagnostic criteria for depression but also because we excluded the effects of medications on brain activity. However, DC does not need to select regions of interest. It can provide objective and comprehensive information on the human-brain connection and characterize the significance of nodes in the network (Chen et al., 2021). Hence, we highlight the increased DC value of the left ITG as a distinctive neuroimaging feature of DPD and a candidate marker to distinguish DPD from NDPD and HC. As a part of the temporal region, ITG, connecting to the hippocampus and other limbic system regions, involves in the evaluation and integration of sensory and emotional information and could be activated by social cognitive tasks (Rive et al., 2013; Schurz et al., 2014). The abnormality of the activation in this region and its connection to the brain regions involved in cognition and emotion lead individuals to focus on negative information. These attention biases subsequently reinforce sadness, deplete mood, and maintain depression (Belzung et al., 2015; Qiu et al., 2021).

The prefrontal cortex (PFC) has a wide range of interconnections with cortical and subcortical structures. Functional changes in this region may lead to abnormalities in the regulation of attention, cognitive control, motivation, and emotion (Gong et al., 2020). The locations of the lesions associated with depression, revealed by previous studies, are highly heterogeneous. However, these lesions mapped to a connected brain circuit centered on the left dorsolateral prefrontal cortex (DLPFC) (Padmanabhan et al., 2019). Gao et al. (2016) observed an increased DC in the left PFC areas in patients with subclinical depression. Another study (Xia et al., 2019) found significantly higher functional activities of the left PFC in MDD than in HC. While several PD studies showed that DPD patients had reduced ALFF in DLPFC (Wen et al., 2013), decreased functional connectivity in the left DLPFC (Lou et al., 2015), and increased connectivity in the DLPFC (Wei et al., 2017), compared to NDPD patients. The present study observed an increase in ReHo and DC in the left DSFG in patients with DPD. More importantly, the ReHo of the left DSFG was positively correlated with the depression scores. The dysfunction of the DLPFC indicates the presence of DPD. However, the exact results are not fully consistent with previous studies. Firstly, ReHo is an indirect measure that reflects the strength of neural activity in the brain and does not directly represent neural activity changes. Whether ReHo is increased or decreased indicates that the neural activity in the local area is synchronization alterations and the region’s collaboration capacity is abnormal (Li et al., 2018). Therefore, the results of this study cannot be directly compared with the above conclusion. Secondly, we completely excluded the effect of medications on the results by recruiting *de novo* non-medicated subjects, which may be one of the reasons for the inconsistency of the results. More importantly, considering the subjects were *de novo* PD patients in this study, the dysfunction of the DLPFC in DPD at the early stage (Braak stage 3) may be secondary to pathology in the dopaminergic projections from the ventral tegmental area to the prefrontal cortex (Starkstein et al., 1989; Ring et al., 1994). The inconsistent disease stages of the subjects may also be responsible for the different results. DSFG is a component of DLPFC, which receives projections from the visual, auditory, and somatosensory cortex, enabling PFC to integrate internal and external information to achieve top-down regulation of attention and affective states (Miller and Cohen, 2001; Kaiser et al., 2015). DLPFC would be active during mental states in which participants attended to emotions or perceptions (Lindquist et al., 2012). Neurons in lateral PFC acquire selectivity for features that reflect specific cue-reward associations (Bichot et al., 1996). We speculate that the abnormal local function and network properties of left DSFG in DPD may underlie the over-perception of negative emotion-related information and abnormal top-down cognitive control over limbic areas (Kaiser et al., 2015), contributing to the generation and development of depression in patients with PD.

### 4.2. Structural alterations in DPD

The amygdala participates in emotional processing, fear conditioning and extinction, motivation, and affective state (Lindquist et al., 2012; Mears and Pollard, 2016). As a densely connected “hub,” the amygdala generates emotional responses to various external stimuli and receives modulations from higher cortices (Huang et al., 2015), implicating its central role in the pathology of depression. Studies focusing on depression had consistently identified structural and functional impairments in the amygdala (Mears and Pollard, 2016). Dopaminergic degeneration in PD induces functional alterations in the amygdala, correlated with the severity of endogenous depression (Sheng et al., 2014). It is reasonable to speculate that the abnormal function of the amygdala may partially contribute to the pathogenesis of the emotional symptoms seen in PD patients. Rs-fMRI studies revealed decreased ReHo and abnormal connectivities of the amygdala in DPD patients, emphasizing the dysfunction of amygdala-related circuits in mood modulation (Sheng et al., 2014; Hu et al., 2015b). Previous studies have found inconclusive findings regarding structural abnormalities in the amygdala in DPD. A study (van Mierlo et al., 2015) reported negative correlations between the score of self-reported depressive symptoms and right amygdaloid volumes in DPD. But other studies demonstrated no amygdala volume difference among DPD, NDPD, and HC (Huang et al., 2015), or no significant correlation between the depressive score in PD and amygdala volume (Bos et al., 2018; Goto et al., 2018). Our study revealed a significant reduction in the volume of the right amygdala in DPD. Heterogeneity of the subjects in age, disease stage, or medication at the time of scan or on prior records is a highly likely source of inconsistency in the results. Other potential sources may include image data processing and statistical correction methods. Overall, the reduction in the amygdala revealed in studies may reflect pathological progression and neuronal death in patients with DPD. In addition, this study also revealed the lateralization of the amygdala to emotional regulation in PD patients. Previously, scholars had pointed out the differences in the function of the amygdala on both sides of the brain (Gläscher and Adolphs, 2003). Consistent with our results, one depression study demonstrated that the structural alterations in the right amygdala are more severe than those in the left (Brown et al., 2020). Following ECT, the right amygdala volume of depression showed a plastic increase (Gryglewski et al., 2019). It follows that structural changes at this site are associated with pathological processes of depression. As emotion modulators, the right amygdala plays a more pivotal role than the left in the occurrence and development of depression (Diederich et al., 2016).

The largest-ever worldwide study by the Enhancing Neuro Imaging Genetics through Meta-Analysis Major Depressive Disorder Working Group reported that adults with MDD have thinner cortical gray matter than controls in several regions, including the temporal lobes (Schmaal et al., 2017). Several meta-analyses also reported a gray-matter loss in the temporal lobe (Arnone et al., 2016) and decreased cortical thickness in the temporal cortex in patients with MDD (Li et al., 2020). Chagas et al. (2017) assessed PD patients with current MDD, lifetime MDD, and no MDD and found a volume reduction in temporal areas in PD patients with lifetime MDD. In agreement with these findings, our study revealed that DPD exhibits a significantly reduced GM volume in the left temporal lobe compared to NDPD and HC. The temporal lobe is involved in language generation and comprehension. Given that complex cognitive tasks of emotional significance often require language and reasoning, the loss of gray matter in the temporal region of depression is not entirely surprising (Arnone et al., 2016).

Although this study revealed a significant reduction in GM volumes in the right amygdala and left temporal lobe compared to NDPD and HC, no correlation was recorded between atrophy and depressive scores. Previous studies (Chagas et al., 2017; Bos et al., 2018; Goto et al., 2018) pointed out the similar characteristics of these findings. Structural abnormalities may vary in different stages of the disease (Goto et al., 2018). Since we recruited *de novo* DPD patients at early stage, the structural damages may not be severe enough to cause significant atrophy corresponding to the severity of depression. Furthermore, the biomarkers of disease presence are distinct from those associated with changes in symptoms over time. Atrophy of the right amygdala and the left temporal lobe might not indicate the existence of depression in *de novo* PD patients. In any case, these structural features deserve further longitudinal analysis in future studies.

The occipital lobe, which contributes to visual information processing and communication with the cerebral cortex, plays a role in facial emotion perception (Teng et al., 2018). Previous studies reported functional abnormalities in the occipital cortex in MDD (Teng et al., 2018) and DPD (Qiu et al., 2021; Wang et al., 2022). Processing bias in affective disorders may initiate as a perceptual visual bias, and attention to negative information may cause and maintain a series of cognitive and affective symptoms, such as depression (Disner et al., 2011; Gong et al., 2020). However, there were rare reports of structural abnormalities in the occipital lobe associated with depression. In this study, the volume of the left occipital lobe was lower in the DPD group than in the HC group. However, there was no significant difference between the patient groups. It remains to be determined whether occipital atrophy is due to neuropathological changes of PD or the pathological mechanisms of depression. We speculate that occipital atrophy is not a specific structural imaging manifestation of depression, at least in patients with *de novo* PD.

### 4.3. MRI indicators in discriminating DPD

This study comprehensively analyzed the role of neuroimaging markers in discriminating DPD from NDPD and HC. After the univariate analysis and correlation analysis, a ROC analysis and the corresponding AUC calculation were applied to evaluate the potential of the ReHo value of the left DSFG, the DC value of the left ITG, and the combined factor as diagnostic markers for the DPD. Our findings depicted nearly the same, good discriminative potential for the ReHo values of the left DSFG and the DC values of the left ITG in the differential diagnosis of DPD. Compared to each of the indicators, the combined factor had a slightly larger power trend in discriminating DPD from NDPD and HC. In particular, the specificity of the combination was higher than that of a simple indicator, which might imply that the complementary use of functional differentiation and integration metrics could be beneficial in screening and differential diagnosis of DPD and merit further investigation.

On the other hand, our results demonstrated that the altered structural indicators fail to survive the correlation analysis and do not further participate in the construction of diagnostic models. Structural and functional alterations were not completely consistent in the present study. Functional disorders explored in this study precede structural atrophy and show greater diagnostic value for DPD. It might mean that functional changes are independent of structural atrophy (Agosta et al., 2012). However, PD patients at different stages have progressive structural alterations (Bai et al., 2021). Considering the enrollment criteria, we speculate that structural atrophy due to pathological impairment in early PD may not be a significant diagnostic marker of depression. Alternatively, the structural alterations are not characteristic indicators of DPD. While further longitudinal follow-up of these structural changes and their relation to depression would be valuable for clarifying the structural characteristics of DPD, this study suggests that fMRI has more potential value in exploring the neuroimaging markers of depression in *de novo* PD.

Several limitations deserve to be mentioned. First, the relatively small number of subjects enrolled in the study may limit the extrapolation of our data to all PD patients. However, stringent inclusion criteria minimized the diagnostic bias. Future large-scale collaborative studies are needed to validate our results. Second, this study remains a cross-sectional study. Longitudinal investigations should be performed by following the subjects to elucidate the functional and structural indicators along the course of PD and to confirm whether these indicators can be used as markers to diagnose and monitor the progression of DPD. Third, the results showed that the abnormal functional indicators with diagnostic significance for DPD are in the left hemisphere. It implies asymmetrical characteristics of pathological lesions in DPD, which is consistent with lateralized characteristics in other neurodegenerative diseases (Minkova et al., 2017). However, we did not consider the side of motor onset and the dominant hand side of the patients in this study. The relationship between the results and the PD lateralization mechanism needs to be explored in future studies. Fourth, we extracted the voxel-based GM volume maps using DARTEL. The TIV, cortical GM volumes, and subcortical GM volumes were obtained based on the VOI segmentation. No significant differences between groups in voxel-based GM volume maps. However, there were differences between groups in the VOI-based cortical and subcortical GM volumes. Different segmentation methods affect the multiple comparison corrections that might contribute to the inconsistency of the results. The pros and cons of the segmentation methods need further analysis to improve the value of sMRI indicators in the DPD differential diagnosis models. In addition, the threshold for computing DC we selected in this study is subjective, although it is consistent with previous studies (Buckner et al., 2009; Wang et al., 2018), Buckner et al. (2009) declared that the selection of different thresholds would have a slight impact on the main results. Future studies focusing on DC at different thresholds are warranted to validate the results of this study.

## 5. Conclusion

To conclude, we propose that both functional and structural impairments exist in DPD and that the ReHo values of left DSFG and the DC values of left ITG and left DSFG are closely related to the severity of depression. More importantly, the above two fMRI indicators and their combination are equally well suited for the diagnosis and differential diagnosis of DPD. Although these results will need to be validated by large-scale clinical cases, it is probably safe to say that the multimodal MRI technique represents a promising approach for the classification of subjects with PD and provides new insight into the neuropathological mechanisms of the disease. More research is needed, for example, on the specific mechanisms of DPD to better understand possible differences between DPD and NDPD regarding neuropathological processes.

## Data availability statement

The original contributions presented in this study are included in the article, further inquiries can be directed to the corresponding author.

## Ethics statement

The studies involving human participants were reviewed and approved by the Medical Research Ethics Committee of Nanjing Brain Hospital. The patients/participants provided their written informed consent to participate in this study.

## Author contributions

WL and HW: conception and study design. HW, JX, GZ, JR, YW, HZ, YS, and JW: data collection or acquisition. HW, JX, MY, and GZ: statistical analysis. WL, HW, JX, and MY: interpretation of results. HW: writing—original draft. WL and MY: writing—review and editing and validation. WL approved the final version of the manuscript to be published. All authors contributed to the article and approved the submitted version.

## Footnotes

1
http://rfmri.org/dpabi

2
http://www.mathworks.com/products/matlab

3
http://surfer.nmr.mgh.harvard.edu

4
https://medcalc.acadtechs.com
