# Supplementary material for: Functional and structural alterations as diagnostic imaging markers for depression in de novo Parkinson’s disease
Source: Front Neurosci. 2023 Feb 22;17:1101623. doi: 10.3389/fnins.2023.1101623 (PMC9992430; doi:10.3389/fnins.2023.1101623)
Supplement: Supplementary file 1 [file Table_1.DOCX]

Supplementary Material

# Supplementary Data

**Table S1** The differences of cortical thickness of PALS_B12 lobes among three groups

| lobes | DPD（n=20） | NDPD（n=37） | HC（n=41） | *F* | *P* value |
| --- | --- | --- | --- | --- | --- |
| left Frontal | 2.39±0.10 | 2.40±0.12 | 2.45±0.12 | 1.365 | 0.261 |
| left Parietal | 2.09±0.08 | 2.10±0.09 | 2.12±0.10 | 0.502 | 0.607 |
| left Limbic | 2.50±0.09 | 2.52±0.08 | 2.54±0.11 | 1.070 | 0.347 |
| left Temporal | 2.62±0.09 | 2.66±0.12 | 2.72±0.12 | 4.117 | 0.019 |
| left Occipital | 1.97±0.07 | 1.95±0.07 | 2.01±0.09 | 4.578 | 0.013 |
| right Frontal | 2.40±0.11 | 2.39±0.11 | 2.43±0.12 | 1.161 | 0.318 |
| right Parietal | 2.07±0.11 | 2.05±0.09 | 2.08±0.09 | 0.851 | 0.430 |
| right Limbic | 2.48±0.07 | 2.51±0.09 | 2.52±0.11 | 0.901 | 0.410 |
| right Temporal | 2.68±0.10 | 2.71±0.11 | 2.76±0.10 | 3.006 | 0.054 |
| right Occipital | 2.01±0.07 | 1.99±0.08 | 2.04±0.10 | 2.453 | 0.092 |

All results of generalized linear models were corrected by Bonferroni for multiple-comparison (*P* -value <0.05/10).
